# Genistein Exerts Anti-proliferative Effects by Regulating Apoptosis and Autophagy-Related Genes and MicroRNAs in Human Urinary Bladder Neoplasm EJ138 Cells: An Experimental and Bioinformatic Study

**DOI:** 10.5812/ijpr-157853

**Published:** 2025-02-22

**Authors:** Alireza Ziyabakhsh, Mohammad Amin Vatankhah, Farid Pakizeh, Ali Nosrat, Pouria Sobhi, Mohammad Vakili Ojarood, Sina Seifimansour

**Affiliations:** 1 Cancer Immunology and Immunotherapy Research Center, Ardabil University of Medical Sciences, Ardabil, Iran; 2 Student Research Committee, School of Medicine, Ardabil University of Medical Sciences, Ardabil, Iran; 3 Department of Radiology, Imam Reza Hospital, Tabriz University of Medical Sciences, Tabriz, Iran; 4 Student Research Committee, School of Medicine, Tabriz University of Medical Sciences, Tabriz, Iran

**Keywords:** Urinary Bladder Neoplasms, Genistein, Apoptosis, Autophagy, MicroRNAs

## Abstract

**Background:**

Bladder cancer (BC) is the most prevalent urogenital malignancy. Recently, the combination of natural compounds with chemotherapeutic agents has gained attention. Genistein, a natural flavonoid, exhibits anti-cancer properties and represents a promising candidate for treating various cancerous cells due to its cytotoxic potential and minimal adverse effects.

**Objectives:**

This study aimed to evaluate the anti-cancer effects of genistein by regulating potential target genes and microRNAs involved in apoptosis and autophagy in EJ138 BC cells.

**Methods:**

EJ138 BC cells were treated with different concentrations of genistein, and cell viability was assessed using the MTT assay. To determine the apoptotic rate of EJ138 BC cells following genistein treatment, flow cytometry with Annexin V/PI staining was performed. Additionally, real-time PCR was conducted to analyze the expression of miR-27a, miR-151, apoptotic genes (caspase-3, caspase-9), and autophagic genes (ATG12, Beclin1) after 48 hours of genistein treatment. Statistical analysis was carried out using SPSS V.22, with independent t-tests and one-way ANOVA. Results were considered statistically significant at P < 0.05.

**Results:**

Our findings demonstrated that genistein inhibited the proliferation, growth, and viability of EJ138 BC cells and induced cell death. Real-time PCR results confirmed that genistein significantly upregulated miR-27a (P < 0.01), ATG12 (P < 0.01), Beclin1 (P < 0.05), caspase-3 (P < 0.001), and caspase-9 (P < 0.0001), while downregulating miR-151 expression (P < 0.05).

**Conclusions:**

The results of this study suggest that genistein suppresses the proliferation and growth of human BC cells by modulating genes and microRNAs involved in apoptosis and autophagy. Therefore, genistein may serve as a novel therapeutic agent for BC treatment.

## 1. Background

Bladder cancer (BC) is the tenth most common cancer in the world, the most common cancer of the urinary system, and ranks thirteenth in lethality among cancers. Its incidence is approximately 550,000 cases annually, accounting for about 3% of newly diagnosed cancers. The incidence and prevalence of BC are increasing, especially in developed countries (1-3). Known risk factors for BC include tobacco use, male gender, genetics, older age, obesity, occupational and environmental toxins (such as aromatic amines), and infections (such as Schistosoma haematobium) (1). The 5-year survival rate for individuals with BC in the United States is approximately 77%, dropping to 5% in metastatic cases. The appropriate treatment approach for BC is chosen based on the tumor's characteristics and may include surgery, radiotherapy, immunotherapy with bacille Calmette-Guérin (BCG), and neoadjuvant or adjuvant chemotherapy (3-5). Despite these treatments, relapse, drug resistance, and lack of response to therapy remain critical challenges in BC treatment (6).

Autophagy and apoptosis are two processes whose dysregulation can lead to the survival of cancer cells. Apoptosis is a programmed cell death process that eliminates unwanted cells through various pathways, involving genes and proteins such as the caspase family. Disturbances in the caspase family and their pathways are crucial in cancer characteristics and drug resistance. Autophagy maintains intracellular homeostasis by digesting defective cell components and oncogenic molecules through molecules such as beclin-1 and ATG. However, autophagy can also support cancer cell survival under certain conditions. Consequently, many studies are exploring new cancer treatment methods by modulating these pathways (7, 8).

MicroRNAs (miRNAs) are short non-coding RNAs, approximately 22 nucleotides long, that play a critical role in post-transcriptional gene expression regulation by binding to target mRNA and degrading it or inhibiting its translation (9, 10). MiRNAs are crucial in regulating apoptosis, cell proliferation, invasion, metastasis, and drug resistance. Different miRNAs can function as tumor suppressors or oncogenes in various cancers, influencing tumorigenesis and treatment outcomes (11). MiR-27a, located on chromosome 19, is involved in proliferation, invasion, apoptosis, cell migration, and chemotherapy drug sensitivity, acting as a tumor suppressor in some cancers, including BC (12). MiR-151, located on chromosome 8, has shown oncogenic properties in various cancers, including hepatocellular, prostate, bladder, and ovarian cancers (13, 14). Targeting these miRNAs with anti-cancer agents offers a promising strategy for BC treatment.

Genistein is a 7-hydroxyisoflavone and natural phytoestrogen found in soybeans, lupin, kudzu, and fava beans. It possesses antioxidant and antineoplastic properties and has demonstrated anticancer activity against various cancers, such as breast, lung, liver, prostate, and kidney cancers, through mechanisms like apoptosis induction, proliferation inhibition, and suppression of angiogenic pathways in various in vitro preclinical studies (15-17). Studies have reported that genistein decreases the expression of miR-21, miR-27a, and miR-221, effectively inhibiting the growth of various cancer cells. Additionally, a study on prostate cancer observed that genistein reduces the expression of miR-151 (14).

## 2. Objectives

This study investigates the effect of genistein on the expression of miR-27a, miR-151, autophagic genes (ATG12, Beclin1), and apoptotic genes (caspase 3, caspase 9) in the EJ138 cell line of BC in vitro.

## 3. Methods

### 3.1. Study Design

Following a literature review and bioinformatic analyses, and based on the results obtained, this in vitro study was conducted. EJ138 BC cells were cultured after being obtained from the Pasteur Institute. These cells were then treated with different concentrations of genistein, and their viability was assessed using the MTT assay. Subsequently, the cells were treated with the pre-determined IC50 concentration of genistein for 48 hours to evaluate the extent of apoptosis and the expression levels of the studied genes and miRNAs using flow cytometry and real-time PCR (RT-PCR), respectively. Untreated EJ138 human BC cells served as the control group in this study.

### 3.2. Bioinformatic Analyses

#### 3.2.1. UALCAN Database Analysis

UALCAN offers an efficient way of analyzing and screening genomic, transcriptomic, and proteomic data associated with a wide range of cancers (18). In this study, the UALCAN database was used to investigate the association between two microRNAs (miRs), namely hsa-miR-151a-5p and hsa-miR-27a, and four genes, including ATG12, BECN1 (BECLIN1), CASP3, and CASP9, in bladder uroepithelial carcinoma (BLCA). The expression levels in normal versus primary tumors and patient survival were investigated in the contexts of these miRs and genes. Additionally, the promoter methylation status of the four genes was also analyzed for the given cancer.

#### 3.2.2. Protein-Protein Interaction Network Construction

Predicted protein-protein interaction (PPI) networks were constructed using the STRING database. STRING utilizes computational methods, text mining, and existing interactions from curated databases to synthesize protein interaction networks (19). The genes were uploaded to the STRING database, and the results were exported to Cytoscape software for further analysis. A combined score of > 0.4 was used for PPI pair extraction.

### 3.3. Chemicals and Reagents

Genistein and MTT were obtained from Sigma-Aldrich. Genistein was dissolved in RPMI before use and stored in a refrigerator at 4°C. The EJ138 human BC cell line was purchased from the National Cell Bank of Iran (NCBI, Pasteur Institute, Tehran, Iran). RPMI-1640 media and fetal bovine serum (FBS) were procured from Gibco. Streptomycin and penicillin were supplied by Sigma-Aldrich.

### 3.4. Cell Culture

EJ138 human BC cell line was cultured in a mixture of RPMI-1640 media, 10% Fetal bovine serum (FBS), 50 μg/mL streptomycin, and 50 units/mL penicillin and were kept at 37°C in a 5% CO2 incubator (Memmert,Germany). In this study, untreated EJ138 human bladder cancer cells were used as the control group. These cells were maintained under identical culture conditions as the genistein-treated groups, without exposure to the compound.

### 3.5. Cell Viability Assay

EJ138 BC cells viability was evaluated by MTT assay. 1 × 10^4^ BC cells were poured into each well of a 96-well plate. Then it was incubated with different concentrations of genistein for 24 and 48 hours. Next, all the wells and cells were washed with PBS, and then 20 µL of MTT solution (5mg/mL) was added to each of the wells, and after 4 hours of incubation, it was replaced by 150 µL of DMSO. Finally, cell viability was measured using an ELISA reader (BioTek,USA) at a wavelength of 570 nm.

### 3.6. Flow Cytometric Analysis for Apoptosis Characterization

To evaluate the extent of apoptosis, 5 × 10^5^ EJ138 BC cells were seeded into each well of a 6-well plate. Then, 39.31 µM genistein (the IC50 value at 48 hours) was added to each well. After 48 hours of treatment, the cells were stained with FITC-conjugated Annexin V and propidium iodide (PI) for 15 minutes. Finally, the cells were analyzed using a flow cytometer to assess the level of apoptosis.

### 3.7. Quantification of microRNAs and Genes Expression by Real Time PCR

To measure the expression of the studied genes, RNAs were extracted and analyzed using real-time PCR. First, the total RNA of EJ138 BC cells (control and treated with the IC50 concentration of genistein) was extracted according to the instructions of the kit used (Invitrogen, USA) and Trizol. Next, 3 μg of RNA was reverse transcribed using the SMOBIO Kit (RP1300, Taiwan) according to the manufacturer's instructions. After primer design using Oligo Primer Analysis Software (version 7.60) (the primer sequences are shown in Table 1), real-time PCR was performed using 2X qPCRBIO SYGreen Mix Lo-ROX (PCRBiosystems, England), and gene expression was evaluated using the RT-PCR detection system (Roche LightCycler 96, Germany). In this study, GAPDH was used as a housekeeping gene, and the 2-ΔΔCt method was used to determine the relative expression of the studied genes. MiR-27a‐5p and miR-151-5p expression levels were calculated using the BON-miR miRNA cDNA Synthesis Kit (Cat: BON209001). The primers used were: miR-27a-5p, 5′-AGGGCTTAGCTGCTTGTGAGC-3′; miR-151-5p, 5ʹ-TCGAGGAGCTCACAGTCTAGT-3ʹ; U6, 5′-AAGGATGACACGCAAA-3′. After polyadenylation, a PCR reaction was conducted in a 10 µL reaction containing 1.0 μL reverse transcriptase (RT) enzyme, 10 μM Bon‐RT primer, 100 mM dNTP mix, and 2.0 μL 10 × RT buffer. The comparative Ct method was used for miRNA expression fold change assessment.

**Table 1.** Sequences of Primers Used for RT-PCR

Genes	Sequences 5’→ 3’
**Caspase 3**	
Forward	ATGGAAGCGAATCAATGGA
Reverse	TGTACCAGACCGAGATGTC
**Caspase 9**	TGTCTACGGCACAGATGGA
Forward	GGACTCGTCTTCAGGGGA
Reverse	
**ATG12**	TTGTGGCCTCAGAACAGTTG
Forward	CCATCACTGCCAAAACACTC
Reverse	
**Beclin1**	CTGGACACTCAGCTCAACGTCA
Forward	CTCTAGTGCCAGCTCCTTTAGC
Reverse	
**GAPDH**	GTCATCCCTGAGCTGAACGG
Forward	CCACCTGGTGCTCAGTGTAG
Reverse	

### 3.8. Statistical Analysis

In this study, SPSS version 22 was used to analyze the data. The statistical significance of the differences was evaluated using an independent *t*-test. A P-value ≤ 0.05 was considered statistically significant.

## 4. Results

### 4.1. Bioinformatic Analysis of miRs

**Figure 1.**
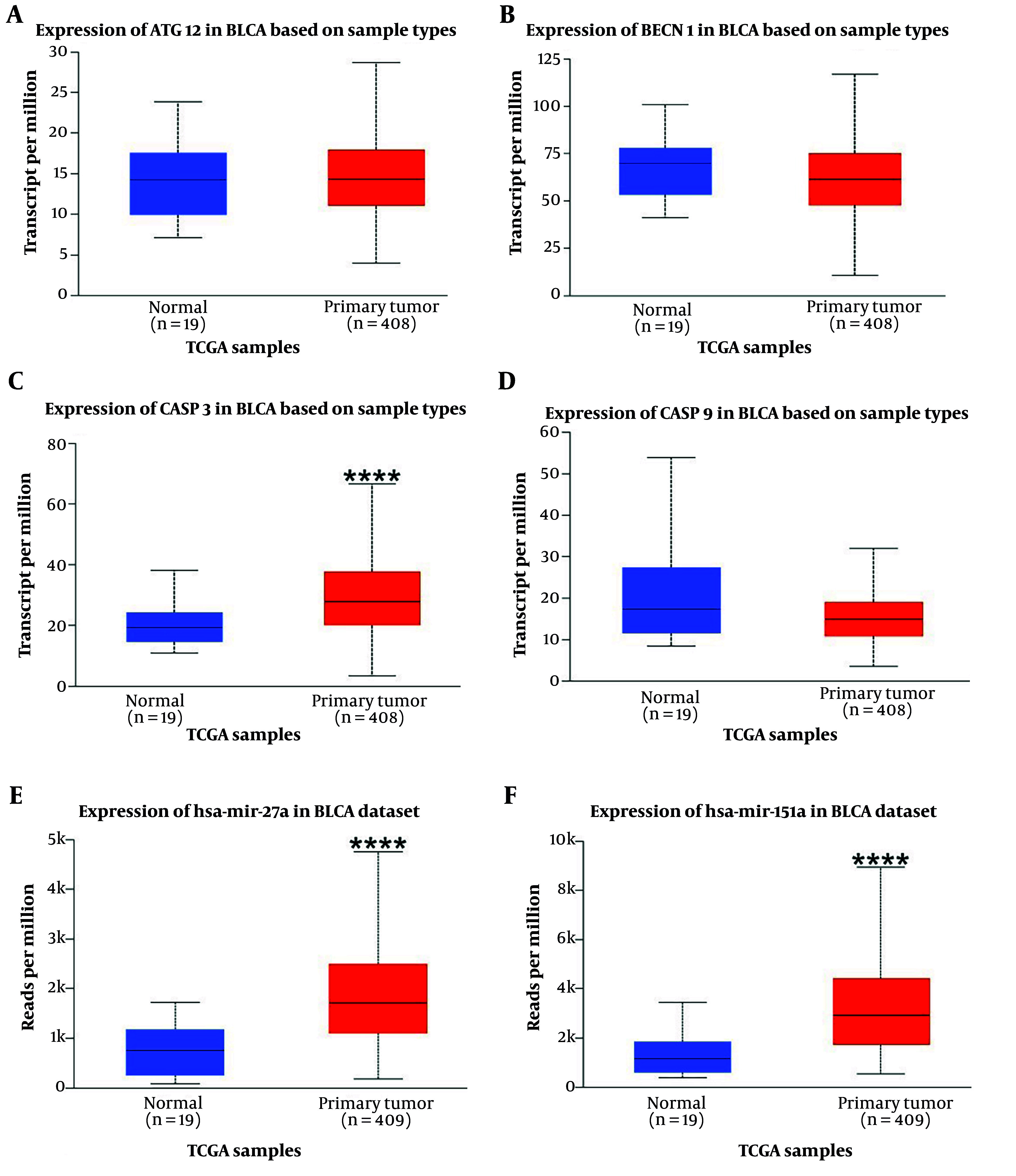
Genes and miRs expression in bladder uroepithelial carcinoma (BLCA) (Bladder Cancer) are shown above in primary tumors vs normal samples based on TCGA (The Cancer Genome Atlas) database samples. **** P-value < 0.0001. A, expression of ATG12 in BLCA based on Sample types; B, expression of BECN1 in BLCA based on Sample types; C, expression of CASP3 in BLCA based on Sample types; D, expression of CASP9 in BLCA based on Sample types; E, expression of miR-27a in BLCA based on Sample types; F, expression of miR-151in BLCA based on Sample types.

**Figure 2.**
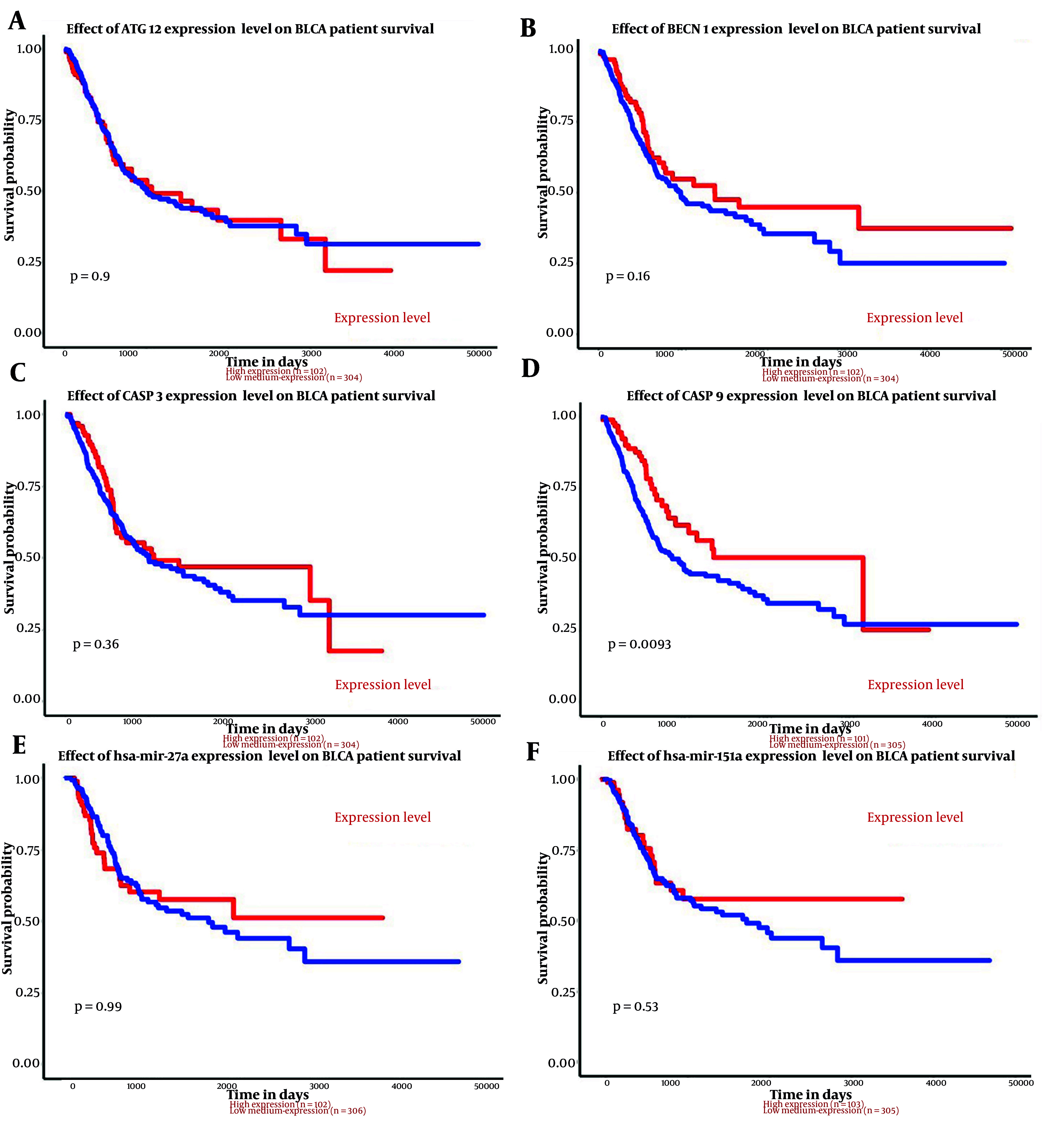
The relationship between genes and miRs expression and survival in bladder uroepithelial carcinoma (BLCA) are shown above. A, effect of ATG12 expression level on BLCA patient survival; B, effect of BECN1 expression level on BLCA patient survival; C, effect of CASP3 expression level on BLCA patient survival; D, effect of CASP9 expression level on BLCA patient survival; E, effect of hsa-mir-27a expression level on BLCA patient survival; F, effect of has-mir-151a expression level on BLCA patient survival.

The analysis results indicated that both miR-151a-5p and miR-27a were significantly upregulated in primary BLCA tumors (n = 409) compared to normal samples (n = 19) (P < 0.0001) (Figure 1). Although higher expression levels of these miRs were associated with an improved survival rate, no significant correlation was observed (Figure 2). 

### 4.2. Bioinformatic Analysis of Genes

**Figure 3.**
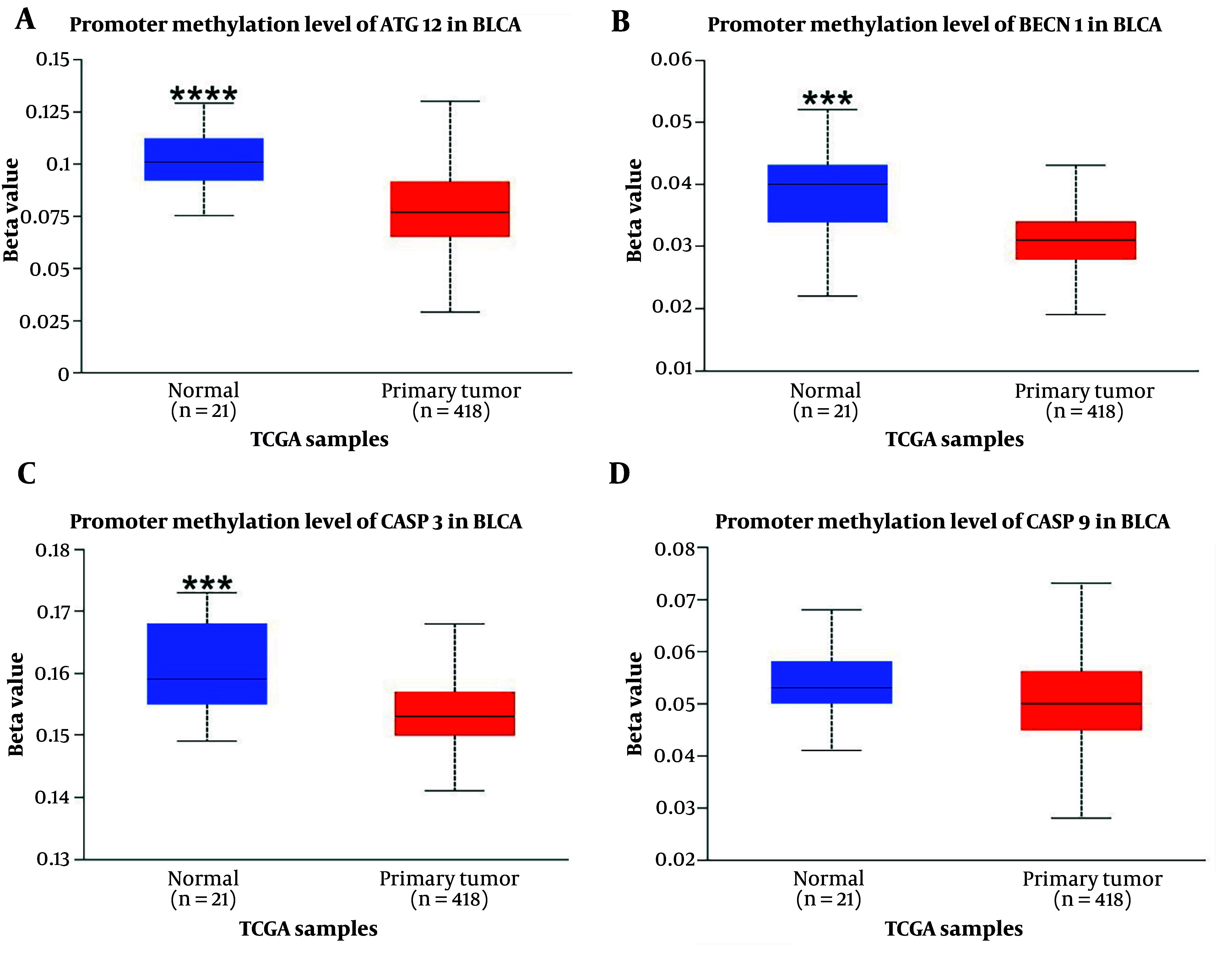
The promoter methylation of genes is shown in bladder uroepithelial carcinoma (BLCA). *** P-value < 0.001; **** P-value < 0.0001. A, promoter methylation level of ATG12 in BLCA, B, promoter methylation level of BECN1 in BLCA; C, promoter methylation level of CASP3 in BLCA; D, promoter methylation level of CASP9 in BLCA.

According to the analysis, of the four input genes, only CASP3 was significantly downregulated in primary tumor samples (n = 418) compared to normal samples (n = 21) (P < 0.0001) (Figure 1). Promoter methylation was significantly higher for ATG12, BECN1, and CASP3 in normal samples as opposed to primary tumors (P < 0.001 for BECN1 and CASP3, P < 0.0001 for ATG12) (Figure 3). Survival analysis found a significant correlation only for CASP9, where patients with higher CASP9 expression demonstrated improved overall survival over time (P < 0.01) (Figure 2). 

### 4.3. Protein-Protein Interaction

**Figure 4.**
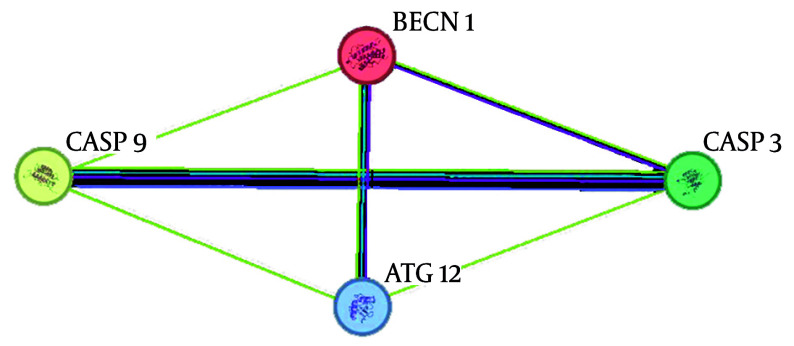
The interaction between genes is shown. The relationship between genes was investigated using Cytoscape Software, using gene fusion, gene coherence, gene neighborhood, experimental studies, text mining, co-expression, curated databases, and protein homology.

We analyzed the four input genes, ATG12, BECN1, CASP3, and CASP9, by connectivity degree in the PPI network. A total of four nodes and six edges were obtained. All genes displayed a node degree of 3 (Figure 4). 

### 4.4. Genistein Induces Cellular Death in EJ138 Bladder Cancer Cells

**Figure 5.**
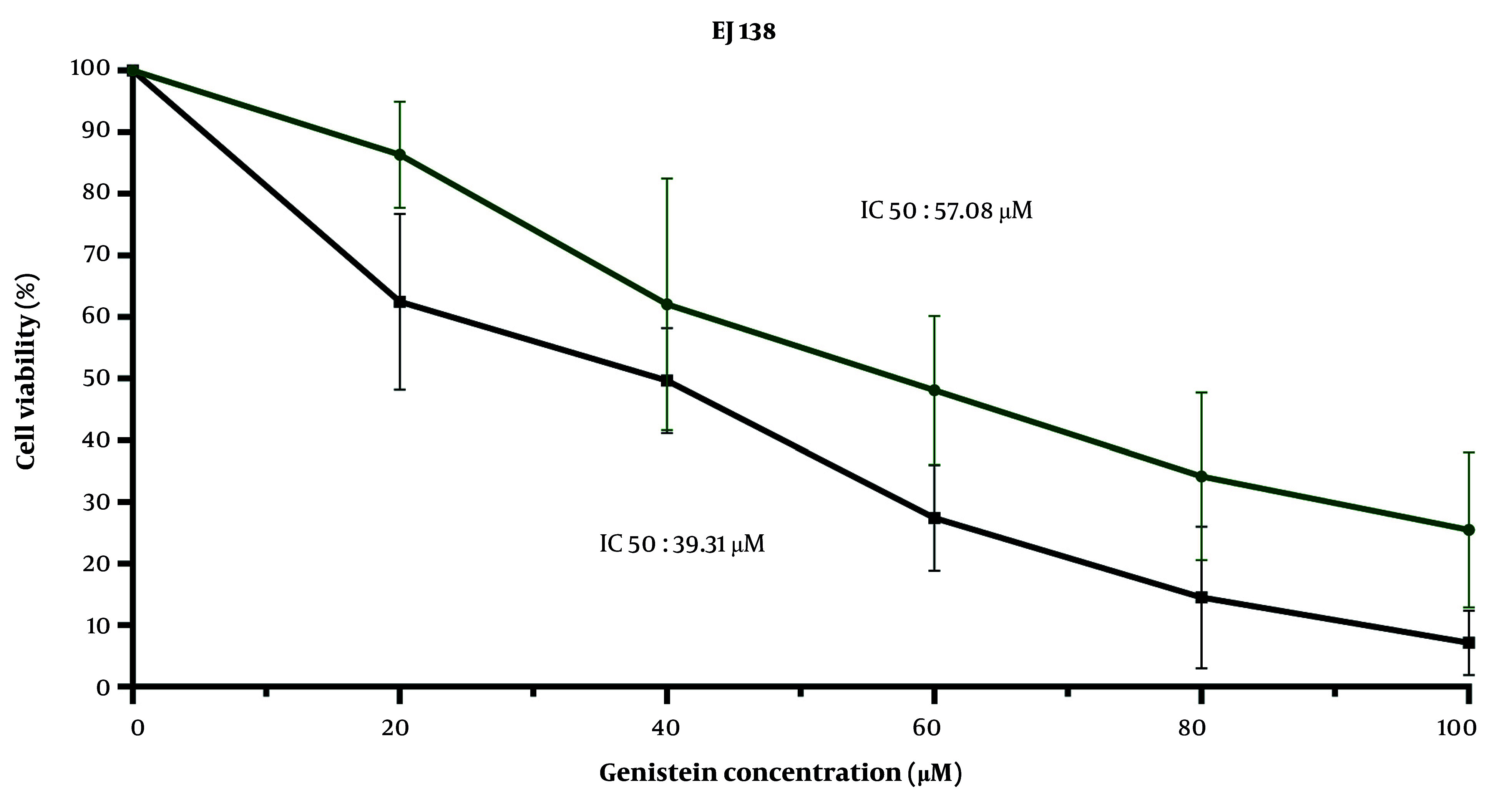
Genistein treatment raises notable cell death in EJ138 bladder cancer (BC) cells. Cell viability analysis of EJ138 BC cells, employing a time- and dose-dependent assay by MTT, was conducted for 24 h and 48 h. 20 - 50 µM genistein was utilized to treat EJ138 BC cells.

According to the results obtained from the MTT assay in this study, genistein decreased cell viability by increasing its concentration from 20 μM to 100 μM in a dose- and time-dependent manner during 24- and 48-hour treatments. The IC50 values of genistein for EJ138 BC cells were 57.08 μM and 39.31 μM after 24 and 48 hours of treatment, respectively (Figure 5). 

### 4.5. Genistein Enhances the Apoptosis Rate in EJ138 Bladder Cancer Cells

**Figure 6.**
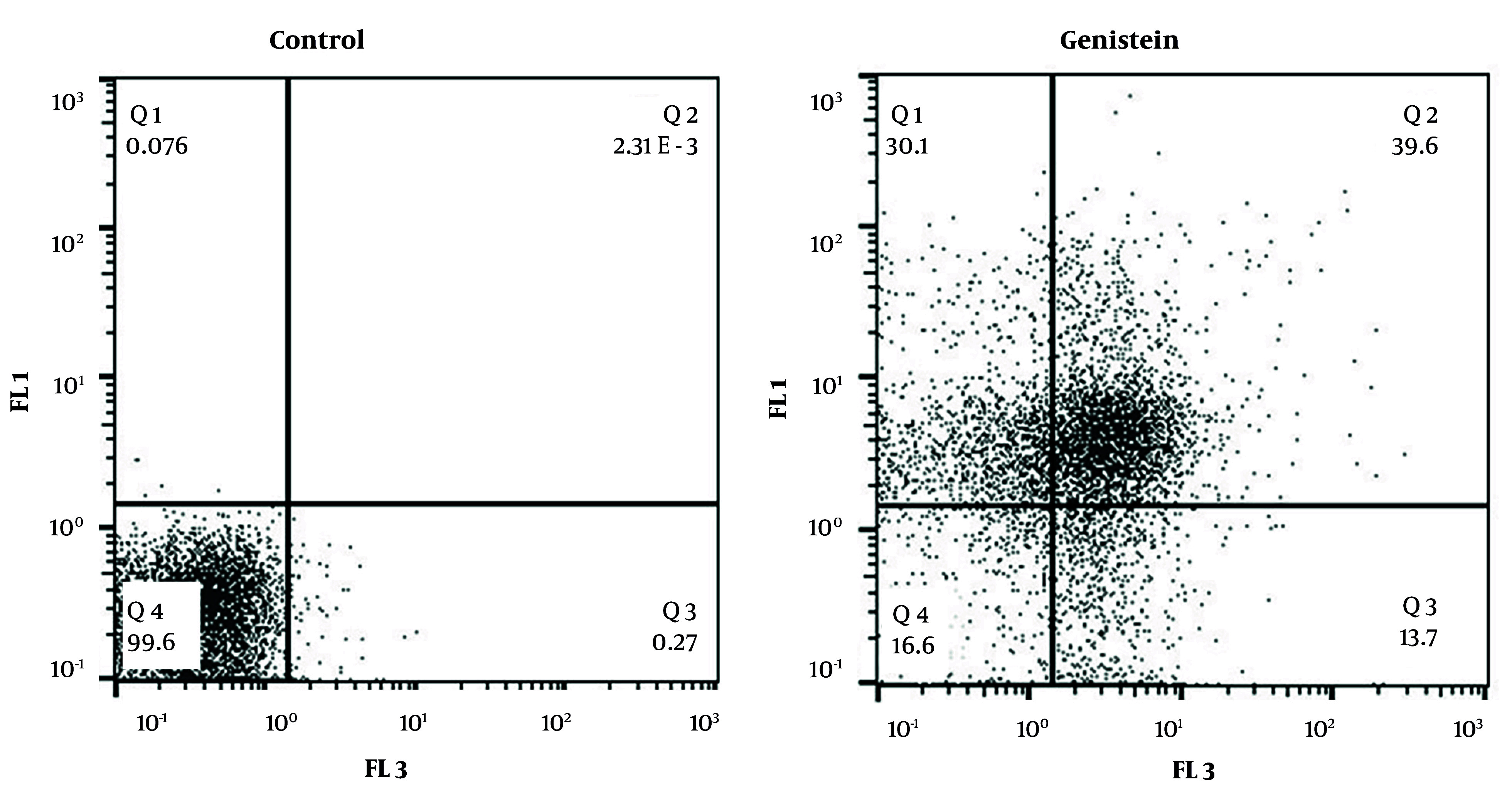
Flow cytometry assay was performed to investigate the effects of genistein on the EJ138 bladder cancer (BC) cells apoptosis after 48 h treatment with 39.31 µM genistein. Q1: Necrotic cells, Q2 and Q3: Apoptotic cells, Q4: Live cells.

According to the results obtained from the treatment of EJ138 BC cells with 39.31 µM genistein (the IC50 value at 48 hours) and subsequent analysis of these samples by flow cytometry, it was observed that genistein increased apoptosis in EJ138 BC cells compared to the control group by 39.6% in quadrant 2 (Q2) and 13.7% in quadrant 3 (Q3) (Figure 6). 

### 4.6. Effectiveness of Genistein in miRNAs and Genes Expression

**Figure 7.**
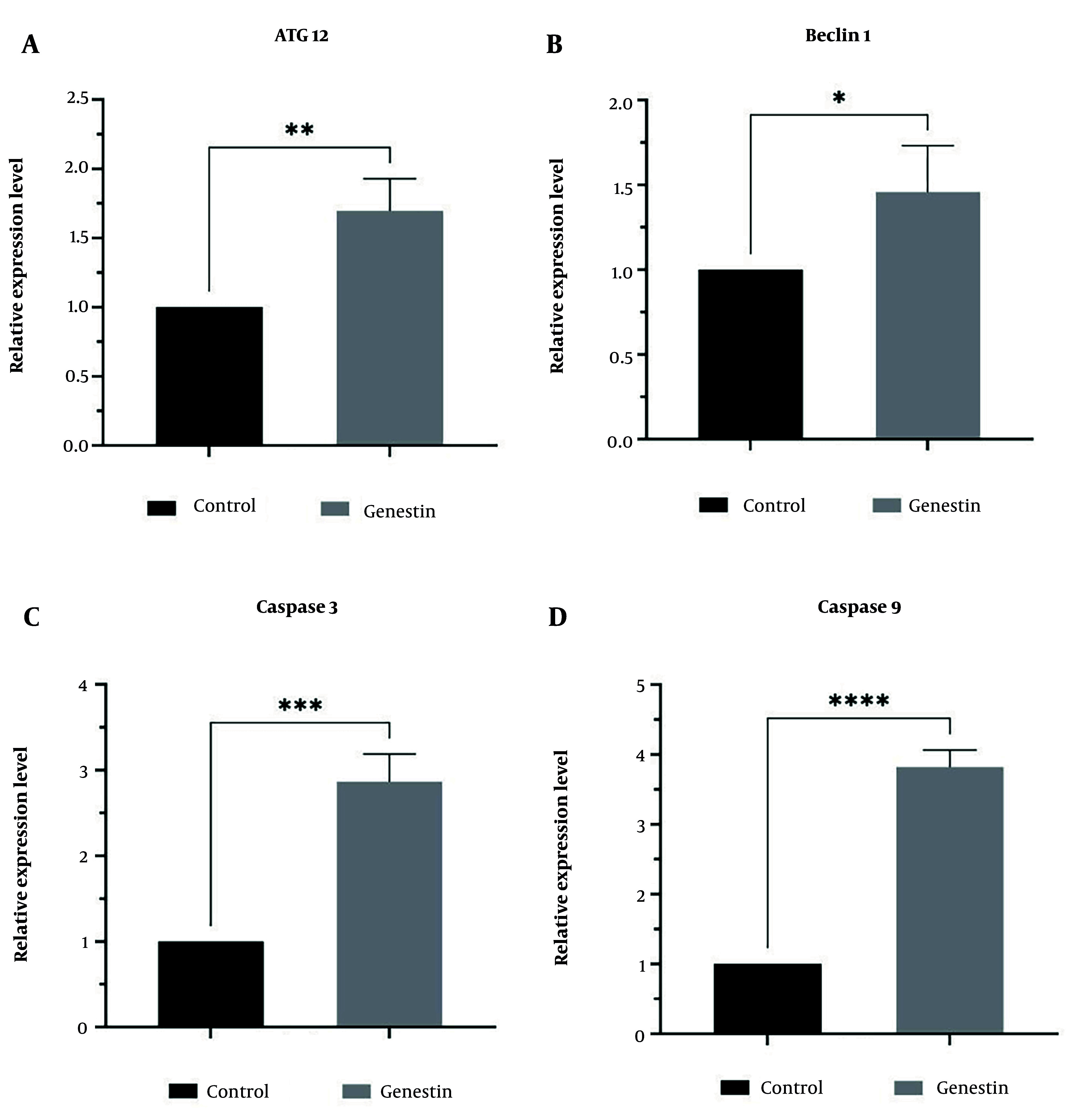
The relative expression of A, ATG12; B, beclin1; C, caspase 3; and D, caspase 9 was assessed by RT-PCR and normalized to GAPDH mRNA level after 48 h treatment of EJ138 bladder cancer (BC) cells with genistein. (* P-value < 0.05, ** P-value < 0.01, *** P-value < 0.001, **** P-value < 0.0001).

**Figure 8.**
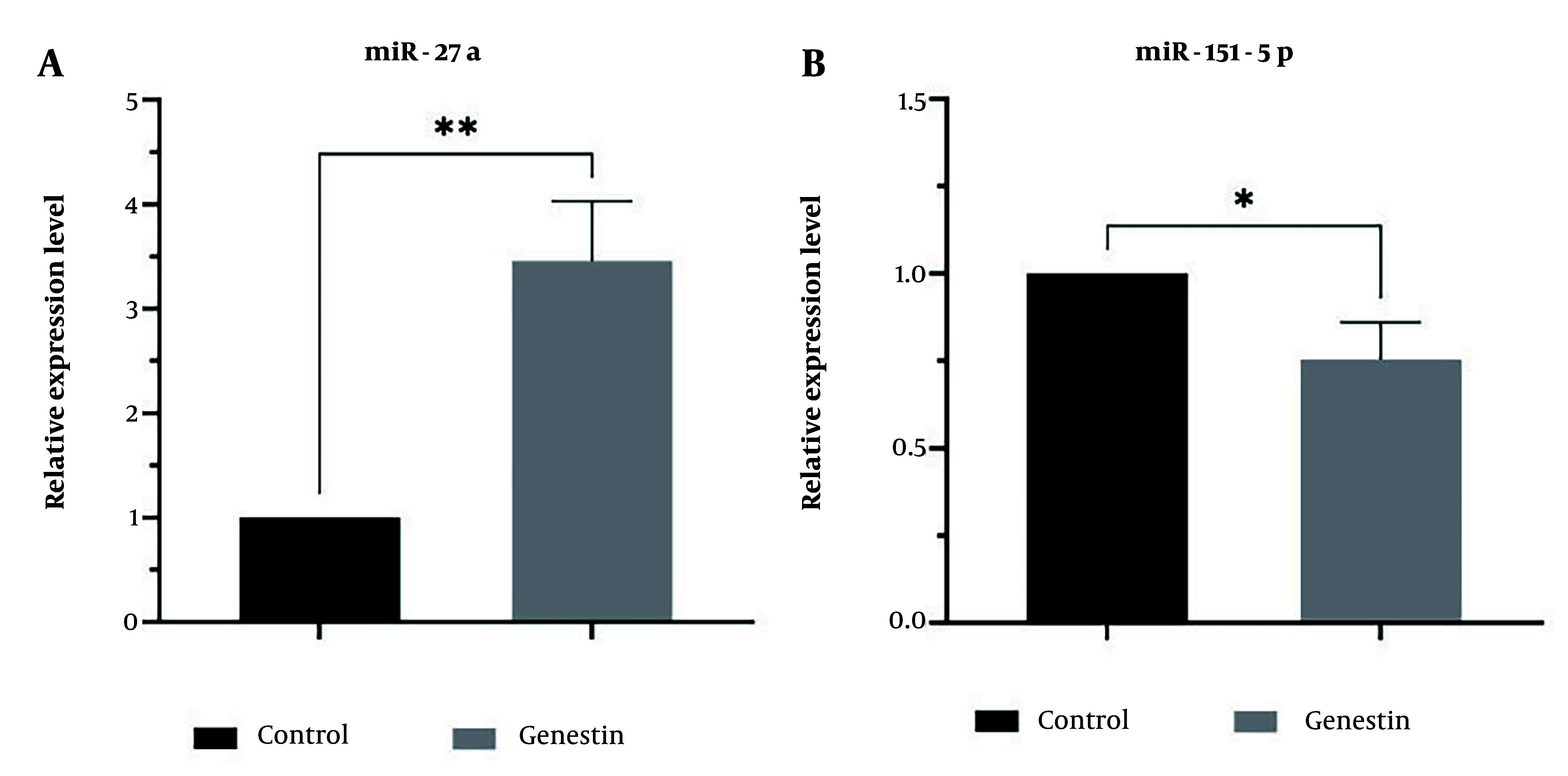
The relative expression of A, miR-27a; and B, miR-151 was evaluated by RT-PCR after 48 h treatment of EJ138 bladder cancer (BC) cells with genistein. (* P-value < 0.05, ** P-value < 0.01).

In this study, the effect of genistein on the expression of miR-27a as a tumor suppressor, miR-151 as an oncogene, and genes involved in apoptosis and autophagy, including ATG12, Beclin1, Caspase 3, and Caspase 9, in EJ138 BC cells was investigated using real-time PCR. According to the results of this study, as shown in Figure 3, genistein increased the expression of miR-27a, ATG12, Beclin1, Caspase 3, and Caspase 9, and decreased the expression of miR-151 in EJ138 BC cells compared to untreated cells (Figures 7 and 8). Therefore, targeting these pathways is at least one of the mechanisms by which genistein induces apoptosis in EJ138 BC cells.

## 5. Discussion

Cancers are diseases whose treatment has been associated with various challenges, such as drug resistance, metastasis, and relapse (20, 21). Therefore, herbal compounds that have fewer systemic side effects than chemotherapeutic agents and the ability to reduce the growth and proliferation of cancer cells by regulating various cellular biological processes can be a novel and hopeful strategy in cancer treatment (22). MicroRNA are RNAs that play an essential role in the expression of various genes, including tumor suppressors and oncogenes, and therefore have critical functions in various cancer characteristics. It seems we can obtain various therapeutic benefits by creating the desired changes in the miRNAs related to each cancer (9-11). The caspase family is a group of proteins that play an important role in apoptosis. Caspase 9 is activated by the apoptosome following events such as DNA breaks and impairment of the cell cycle, and then, by activating effectors such as caspase 3, it causes the breakdown of cellular proteins and induces apoptosis. Beclin1 is a protein that acts as a scaffold and, together with some other particular proteins, forms the Class III PI3K complexes, which ultimately play an important role in autophagy by forming phosphorylated phosphatidylinositol (23, 24). ATG12 is a protein that, after being activated by ATG7, binds to ATG5 and forms a complex that then interacts with ATG16L to form the larger ATG12-ATG5-ATG16L complex, which plays a key role in autophagosome formation and the occurrence of autophagy (25). Therefore, investigating and targeting these pathways can lead to the discovery of novel cancer treatments.

In this study, we evaluated the effect of genistein on the cell viability and apoptosis of EJ138 cells. It was observed that genistein decreases the viability and increases apoptosis of these malignant cells. We also investigated the effect of genistein on the expression of miR-27a and miR-151, as well as autophagic and apoptotic genes, including ATG12, Beclin1, caspase 3, and caspase 9, in the EJ138 cell line of BC in vitro. Based on the results of this study, genistein decreased the expression of miR-151, an onco-miR in BC, and increased the expression of miR-27a, a tumor suppressor in BC. An increase in the expression of ATG12, Beclin1, caspase 3, and caspase 9 genes, which are among the genes involved in cell autophagy and apoptosis, was observed. Thus, at least one of the proposed mechanisms in the increase of apoptosis caused by genistein in EJ138 cells is due to the effects of this isoflavone on miR-27a and miR-151. However, other mechanisms may also be involved in increasing apoptosis and decreasing the viability of these malignant cells, which will require further studies.

In a study conducted on the effect of genistein on retinoblastoma, it was observed that genistein reduces the expression of ABCE1 by inducing the expression of miR-145, ultimately inhibiting the growth of cancer cells (26). In another study, it has been reported that genistein increases the expression of tumor suppressors sFRP1 and Smad4 in prostate cancer cells by reducing the expression of miR-1260b and also DNA methylation (27). Genistein can also downregulate the expression of miR-151, which is an oncogene in prostate cancer cells (14), and upregulate miR-27a expression in A549 lung cancer cells, inhibiting the proliferation of these cancerous cells (28). In another study, it was observed that genistein decreases the expression of miR-223 and the growth of prostate cancer cells (29). In a study conducted by Xia et al., they revealed that genistein could increase the expression of miR-34a and thereby downregulate the Notch-1 signaling pathway, ultimately leading to the inhibition of proliferation and growth of prostate cancer cells and inducing apoptosis in them (30). Zaman et al. showed that genistein could inhibit miR-23b-3p expression, which is an oncomiR that suppresses the PTEN tumor suppressor gene in renal cancer cells (31). de la Parra et al. demonstrated that genistein induces apoptosis and decreases cell viability in breast cancer cells, and according to this study, this effect can be through reducing the expression of miR-155, which is an oncogene (32). In 2020, Chen et al. revealed that genistein reduced cell viability and increased apoptosis in colorectal cancer cells by inhibiting miR-95/SGK1/Erk1 expressions (33).

### 5.1. Conclusions

According to the results obtained from this study, genistein induces apoptosis and inhibits the proliferation of EJ138 BC cells by upregulating the expression of miR-27a, apoptosis-related genes (caspase 3 and caspase 9), and autophagy regulator genes (ATG12, Beclin1), while downregulating miR-151 expression. Based on these promising results, genistein may have potential therapeutic effects in BC, which warrants further studies.

## Data Availability

The dataset presented in the study is available on request from the corresponding author during submission or after publication.
